# Resection of a craniopharyngioma using the "inside-out" technique: a two-dimensional operative video

**DOI:** 10.3171/2026.7.FOCVID26115

**Published:** 2026-10-01

**Authors:** Zhengyuan Chen, Benjamin J. Carnovale, Adway Gopakumar, Shovan Bhatia, Garret Choby, Eric W. Wang, Carl H. Snyderman, Georgios A. Zenonos

**Affiliations:** 1 Department of Neurological Surgery, Center for Cranial Base Surgery, University of Pittsburgh Medical Center, Pittsburgh; and; 2 Department of Otolaryngology, Center for Cranial Base Surgery, University of Pittsburgh Medical Center, Pittsburgh, Pennsylvania

**Keywords:** craniopharyngioma, endoscopic endonasal approach, inside-out technique, subpial dissection technique

## Abstract

A 57-year-old man presented with headache and obstructive hydrocephalus caused by a craniopharyngioma. He underwent gross-total resection through an endoscopic endonasal approach. The key surgical concept was the "inside-out" technique, in which the tumor was internally decompressed first, allowing the capsule to collapse and the dissection plane to be developed gradually from the center of the lesion toward its peripheral pial interface. This helped limit traction on the optic apparatus, hypothalamus, and perforating vessels. Multilayer reconstruction was performed and assessed using indocyanine green fluorescence endoscopy. This video highlights the technical nuances of the inside-out technique for maximal safe resection of craniopharyngiomas.

The video can be found here: https://stream.cadmore.media/r10.3171/2026.7.FOCVID26115

## Transcript

This video will demonstrate the "inside-out" technique in the resection of craniopharyngiomas.^1^

### 0:25 Clinical Presentation.

The case refers to a 57-year-old man who presented with confusion, drowsiness, and imbalance, resulting in a fall. He had unrelenting headaches over the past several months, and he had not seen physicians. He did have an alcohol use disorder as well as he smoked heavily.

### 0:39 Physical Examination.

He was drowsy but arousable. He was oriented but had difficulty with higher-order executive functions and memory. He did have subjective blurred vision, but his visual fields were normal to confrontation, and he was intact otherwise.

### 0:52 Neuroimaging Findings and Preoperative Course.

Imaging disclosed a suprasellar, contrast-enhancing lesion, causing obstructive hydrocephalus with significant extension up to the third ventricle, and he did have internal calcifications, which were suggestive of an adamantinomatous craniopharyngioma. An external ventricular drain was placed.

### 1:09 Surgical Approach Considerations.

Because of the obstructive hydrocephalus, surgical resection was recommended. Whenever considering resection for craniopharyngiomas, a heavily T2-weighted image is obtained to evaluate the suprasellar window. You see here the optic nerve in red and the suprasellar window in the yellow arrows. When considering approach selection, anterolateral and posterolateral approaches, we believe, have a poor visualization of the ipsilateral interface with the hypothalamus. The interhemispheric transcallosal transventricular approach is a little limited because it is tethered by the callosotomy, providing limited versatility, and similarly the interhemispheric translamina terminalis has a blind aspect right behind the chiasm. An endoscopic endonasal approach aligns perfectly with the long axis of the lesion and provides visualization of both interfaces with the hypothalamus.^2,3^

### 1:59 Patient Positioning and Setup.

The patient was positioned supine and was placed in three-point fixation. We did use stereotactic neuronavigation. We had a Doppler available and diamond burrs.

### 2:09 Key Operative Steps.

The key surgical steps of the procedure consist of the nasal step, where we raise the nasoseptal flap and a contralateral reverse flap; the bony exposure, exposing the tuberculum and planum; followed by the inside-out technique, which we will analyze; and, of course, the multilayer reconstruction.^4^

### 2:25 The Inside-Out Dissection Technique.

It is notable that craniopharyngiomas are intra-axial lesions that displace the pia and its microvasculature centrifugally.^5^ This is in contrast to suprasellar lesions that are extra-axial, such as meningiomas, which displace the microvasculature posteriorly. So the microvasculature of the hypothalamus runs in the pia and in classic craniopharyngioma surgery, with subarachnoid dissection. Subsequently, this cannot be extrapolated up toward the third ventricle, and rather, a sharp dissection of the pia has to ensue at the level of the hypothalamus, which potentially disrupts the microvasculature.^6^ In contrast, we believe an epi-tumoral dissection plane or a subpial dissection plane facilitates extrapolation of this plane of dissection into the third ventricle and preserves the microvasculature that you see here in red. We believe that the correct dissection plane for this craniopharyngioma is epi-tumoral and subpial, but not subarachnoid. So, this inside-out technique consists of finding where the tumor comes closer to the surface and establishing an entry into the tumor and start dissecting the tumor from the inside-out, debulking the tumor internally, and starting dissecting the tumor in a subcapsular fashion, just above the tumor, resulting at the end with the pia staying intact. So, the tumor dissection is not subarachnoid, but epi-tumoral or subpial.

### 3:54 Endoscopic Endonasal Sellar Exposure and Durotomy.

This is the demonstration of the procedure. We start with a wide sphenoidotomy. We already raised a right-sided nasoseptal flap and performed a reverse flap. This is performed in conjunction with ENT and neurosurgery with dynamic endoscopy. We perform a wide exposure, exposing widely the sella and removing the middle clinoids as well as the medial opticocarotid recesses. We proceed to expose the tuberculum and some of the planum. We also expose the floor of the sella, and this facilitates downward displacement of the diaphragma, which increases our suprasellar space and working window. The exposure of the planum is not absolutely necessary, but for tall tumors, it does facilitate superior displacement of the instruments. But certainly, no dural opening is necessary in the planum. We open above and below the superior intercavernous sinus, which was coagulated and divided, followed by exposure of the suprasellar space in the prechiasmatic recess. You see here the microvasculature that’s running in the pia.

### 5:12 Internal Tumor Debulking.

We do not perform the extensive subarachnoid dissection, but rather open into the tumor and start internally debulking it to create space. The next step is to start to create this epi-tumoral dissection plane. You see here we’re not dissecting the tumor in the subarachnoid space, but rather create a space within the tumor after internal debulking and intracapsular dissection.

### 5:48 Subpial Dissection Plane.

So, we’re staying right above the tumor and start developing this dissection plane right above the tumor. We’re not in the subarachnoid space; we’re in the subpial space. This is a back-and-forth between internal debulking and intracapsular dissection. You see how nicely the pia stays with its microvasculature and is not disrupted. At no point here we’re cutting sharply at the interface with the hypothalamus, but rather, we’re trying to stay right under the tumor. Again, back-and-forth internal debulking.

### 6:30 Tumor Resection and Delivery.

And usually, the attachment of the tumors, the craniopharyngiomas, are at the tuber cinereum or the stalk. So once these are dissected, the tumor can be nicely delivered. You can see here we did not disrupt the microvasculature of the hypothalamus, and the surrounding pia with its microvasculature remains intact.^5^

### 6:53 Multilayered Skull Base Reconstruction.

Reconstruction of course is very important. We place a dural substitute followed by our nasoseptal flap, which is nicely enhancing with ICG angiography. We placed some Surgicel and packing we already have^7^ CSF diversion with an external ventricular drain in this case.

### 7:17 Postoperative Course.

So, the patient’s EVD was weaned. Unfortunately, he did have pulmonary embolism requiring anticoagulation, but he remained neurologically intact. His mental status improved and was discharged to rehabilitation. He required both DDAVP and hydrocortisone replacement. Of note, the preservation of the stalk with this inside-out technique does not necessarily always aim at preservation of function, but rather, it aims at preserving the correct dissection plane and extrapolating up toward the third ventricle for a more complete resection and preservation of the correct planes.

### 7:52 Postoperative Radiographic Outcomes.

Here is his immediate postoperative scan, showing no immediate complications and a complete resection. And here, the review of the 9-month MRI, at which point the patient was completely neurologically intact with no residual or recurrence.

### 8:10 Conclusions.

So, in conclusion, craniopharyngiomas are intra-axial tumors. In our opinion, the correct plane of dissection is epi-tumoral, subpial, and not subarachnoid. Microvascular injury can lead to morbidity and hypothalamic injury and visual disturbances, and this inside-out technique can potentially decrease microvascular injury and facilitate the correct plane of dissection with the hypothalamus and the third ventricle.^7^

## Disclosures

The authors report no conflict of interest concerning the materials or methods used in this study or the findings specified in this publication.

## Author Contributions

Primary surgeon: Zenonos. Assistant surgeon: Choby, Wang, Snyderman. Editing and drafting the video and abstract: Chen, Carnovale, Gopakumar, Bhatia, Choby. Critically revising the work: Zenonos, Chen, Gopakumar, Bhatia, Choby, Wang, Snyderman. Reviewed submitted version of the work: all authors. Approved the final version of the work on behalf of all authors: Zenonos. Supervision: Zenonos, Chen, Choby.

## Supplemental Information

### Patient Informed Consent

The necessary patient informed consent was obtained in this study.
